# De novo *ADGRV1* variant in a patient with ictal asystole provides novel clues for increased risk of SUDEP

**DOI:** 10.1186/s42494-023-00124-5

**Published:** 2023-05-24

**Authors:** Tuo Ji, Aaron W. Downs, Luong Dorris, Ning Zhong

**Affiliations:** 1 SQZ Biotechnologies, Watertown, MA 02472 USA; 2 Morrissey College of Arts and Sciences, Boston College, Chestnut Hill, MA 02467 USA; 3 College of Medicine, California Northstate University, Elk Grove, CA 95757 USA; 4 Pediatric and Adolescent Medicine Residency of Mayo Clinic, MN Rochester, USA; 5 Department of Neurology, Kaiser Permanente Sacramento Medical Center, Sacramento, CA 95825 USA; 6 North Valley Comprehensive Epilepsy Program, Kaiser Permanente Sacramento Medical Center, Sacramento, CA 95825 USA

**Keywords:** Ictal asystole, Drug resistant epilepsy, ADGRV1, SUDEP

## Abstract

**Background:**

Various cardiac and autonomic manifestations are frequently reported during seizures. Among the seizure-related arrhythmia, ictal tachycardia is the most common, followed by ictal bradycardia, with ictal asystole being the rarest. The occurrence of ictal asystole may obscure the clinical presentation and delay the diagnosis, representing a life-threatening presentation of epilepsy, with an elevated risk of sudden unexpected death in epilepsy patients (SUDEP). These cardiac abnormalities are being increasingly recognized as the key to elucidating the mechanisms of SUDEP.

**Case presentation:**

We present a 35-year-old man with a history of focal-onset seizures with impaired consciousness since his mid-20 s. He developed different types of seizures for 2 years, described as tonic seizure and atonic seizure (drop attack). During such clinical events, he suffered from falls and cardiac arrest. However, thorough cardiac electrophysiology and imaging workup failed to reveal a cardiac etiology. Subsequent video electroencephalograph (EEG) monitoring was performed, and ictal bradycardia and ictal asystole were discovered. A cardiac pacemaker was implanted, and at 3-year follow-up, the patient did not suffer more atonic seizures, or falls. Genetic tests discovered a de novo variant of Adhesion G Protein-Coupled Receptor V1 (*ADGRV1*), which may provide a clue for the patient’s ictal asystole and the increased risk of SUDEP.

**Conclusions:**

Considering the important impact of ictal bradycardia and asystole on the morbidity and potential mortality of epileptic patients, it is important to simultaneously utilize EEG and electrocardiogram to confirm the diagnosis. This case report highlights the link between the de novo variant of *ADGRV1* and the ictal bradycardia/asystole phenotype and implicates the importance of genetic testing in adult epilepsy patients.

## Background

Epileptic seizure-induced cardiac arrhythmias have long been recognized as epileptic ictal manifestations, possibly caused by seizure-induced autonomic imbalances [1]. The most common arrhythmia associated with epilepsy is ictal tachycardia, which occurs in ~ 80% of all seizures. Ictal bradycardia occurs in < 6% of seizures. Ictal asystole, the absence of ventricular complexes for more than 4 s, accompanied by electrographic seizure onset, is found in 0.27–0.4% of patients undergoing video-electroencephalograph (vEEG) monitoring [2]. Ictal bradycardia/asystole is often unrecognized until documented during vEEG–electrocardiogram (ECG) monitoring in drug-resistant epilepsy (DRE). As lethal arrhythmias during seizures, ictal ventricular tachycardia, ictal bradycardia, and ictal asystole, are hypothesized as among the pathophysiological causes of Sudden Unexpected Death in Epilepsy Patients (SUDEP) [3].

SUDEP is a sudden, unexpected, witnessed, or unwitnessed death in an individual with epilepsy that is not caused by a traumatic injury, drowning, or other causes. SUDEP occurs in benign circumstances with or without evidence for a seizure and excludes documented status epilepticus. Postmortem examination does not reveal other causes of death [4]. However, at present, there is no consensus on the causes of ictal bradycardia/asystole and their effect on the pathophysiology of SUDEP. The mechanisms of ictal bradycardia/asystole might be explained by the associations between the central autonomic network control and the limbic system structures such as the cingulate gyrus, amygdala, and insular and orbitofrontal cortex [5]. However, such hypothesis does not entirely account for the various clinical manifestations and brain structural lesions documented. Recent studies are pointing to the genetic susceptibility to SUDEP. A few variants of genes involved in epilepsy as well as cardiac and respiratory functions have been discovered, suggesting a highly heterogenic and polygenic contribution to SUDEP [6]. Adhesion G protein-coupled receptor V1 (ADGRV1) has been implicated in the regulation of breathing and cardiovascular function, and recent studies have suggested that it also plays a role in SUDEP.

This is in line with the increasing efforts to practice precision medicine in epilepsy. Based on genetic information, therapies such as anti-seizure medication can be tailored to achieve the best therapeutic efficiency while minimizing side effects and intolerance, ultimately leading to the development of effective, personalized gene therapies [7].

## Case presentation

We report a 35-year-old Caucasian man with DRE who suffered from ictal asystole. Clinical presentation, brain imaging, EEG data, genetic testing results, and treatment outcome were reported.

### Clinical characteristics

The 35-year-old, right-handed male had a history of focal seizures with impaired consciousness initially diagnosed at age 24 in 2010. Semiology by then was described as difficulty in speaking, incomprehensible speech, staring spells, behavioral freeze, and impaired awareness. Such episodes lasted from 30 s to minutes. Postictally, the patient was amnestic of the event and only spoke in his native language. The post-ictal state lasted approximately 10 min. Based on the described semiology, the patient was diagnosed as focal onset seizures with impaired awareness (also known as complex partial seizures). He was prescribed Levetiracetam, and the dose was increased according to the reported recurrent seizures. An EEG during this period showed mild generalized slowing in the theta range, though no epileptiform features were identified. Brain magnetic resonance imaging (MRI) in 2013 did not reveal any abnormality.

In 2015, he was presented to the Emergency Department (ED) after a witnessed generalized tonic–clonic seizure (GTCS) secondary to not-taking medication. Nonetheless, Levetiracetam alone failed to control the patient’s seizures as the seizure frequency continued to increase to once daily or multiple times per day. Then lamotrigine was added to his regimen and the dosage was eventually maximized. Consequently, seizure control was maintained for 2 years until a recurrent GTCS in 2017. Follow-up EEG showed frequent left-sided slowing and slow sharp waves at C3T3, and occasional right-sided temporal sharp-waves.

A few months later, new seizure semiology developed. The patient was observed to have (1) tonic seizures, with witnessed body stiffening, lasting 1–2 min; (2) atonic seizures/drop attack, with witnessed falls and loss of consciousness; and (3) nocturnal brief myoclonic events. The patient had been admitted to the ED on multiple occasions after atonic events. During one incident, he fell backwards onto a concrete floor resulting in a small left-sided subarachnoid hemorrhage and temporo-occipital skull fracture. There was no witnessed tonic–clonic activity proceeding the fall. As the patient’s seizures resulted in more debilitating consequences, Lacosamide was introduced. However, no clinical improvement was observed. In April 2019, he was witnessed to have full-body stiffening and shaking before losing consciousness. In the field, when paramedics arrived, he was found in pulseless cardiac arrest. He showed spontaneous restoration of normal sinus rhythm before any interventions were administered. Cardiac workup including ECG, trans-esophageal echocardiogram, stress ECG test, and electrophysiology yielded no findings indicative of a primary cardiac etiology for his cardiac arrest.

### Video EGG monitoring

**Fig. 1:**
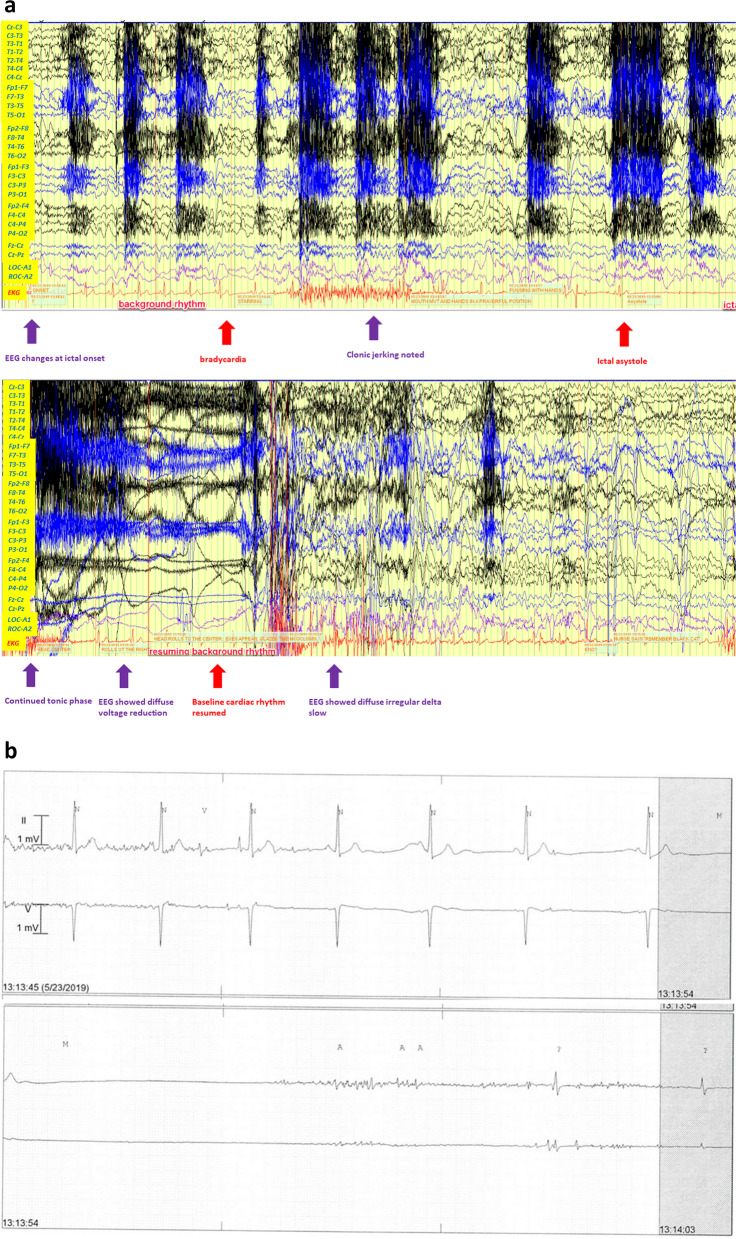
**a** Atonic seizure recorded during video EEG (vEEG) monitoring. vEEG showed ictal generalized delta slowing, followed by tonic (stiffening) and clonic (jerking) phases; and subsequent diffuse voltage attenuation and diffuse distribution of delta slowing (purple arrows). The one lead ECG recording (red lines in the figure) showed HR change (red arrows) when bradycardia and asystole; **b** Ictal asystole was further confirmed by the ECG traces recorded during cardiac telemetry monitoring

The patient continued to suffer recurrent seizures despite taking three antiseizure medications at sufficient dose, so he was diagnosed with DRE. VEEG was performed in May 2019. Ictal bradycardia and ictal asystole were observed. During one of his typical seizures, he was observed with frozen behavior and staring; then, he exhibited hand fidgeting with fingers locked in tonic posture. His body proceeded to fall back to bed, with his head turning to the left accompanied by mild non-rhythmic limb shaking. Subsequently, the patient lost consciousness, followed by myoclonic body jerking. EEG showed diffuse, irregular delta slowing at the onset of the event. Such diffuse slowing persisted throughout the 13.5 s of asystole. The asystole was also confirmed with concurrent cardiac-telemetry monitoring. During the asystole, EEG showed diffuse low voltage recording, indicating global cerebral hypoperfusion (Fig. 1a). The patient experienced four similar seizures when bradycardia and shorter asystole (heart rate pauses for a few seconds) were captured in EEG-ECG monitoring (Fig. 1b). The onset of bradycardia and heart-beat pauses were noted 20 s after the seizure onset (Fig. 1a). Cardiology was consulted and the patient underwent emergency cardiac pacemaker implantation to prevent further cardiac complications in DRE. The pacing parameter was set at 60–130 bpm.

### Follow-up and treatment outcome

**Fig. 2:**
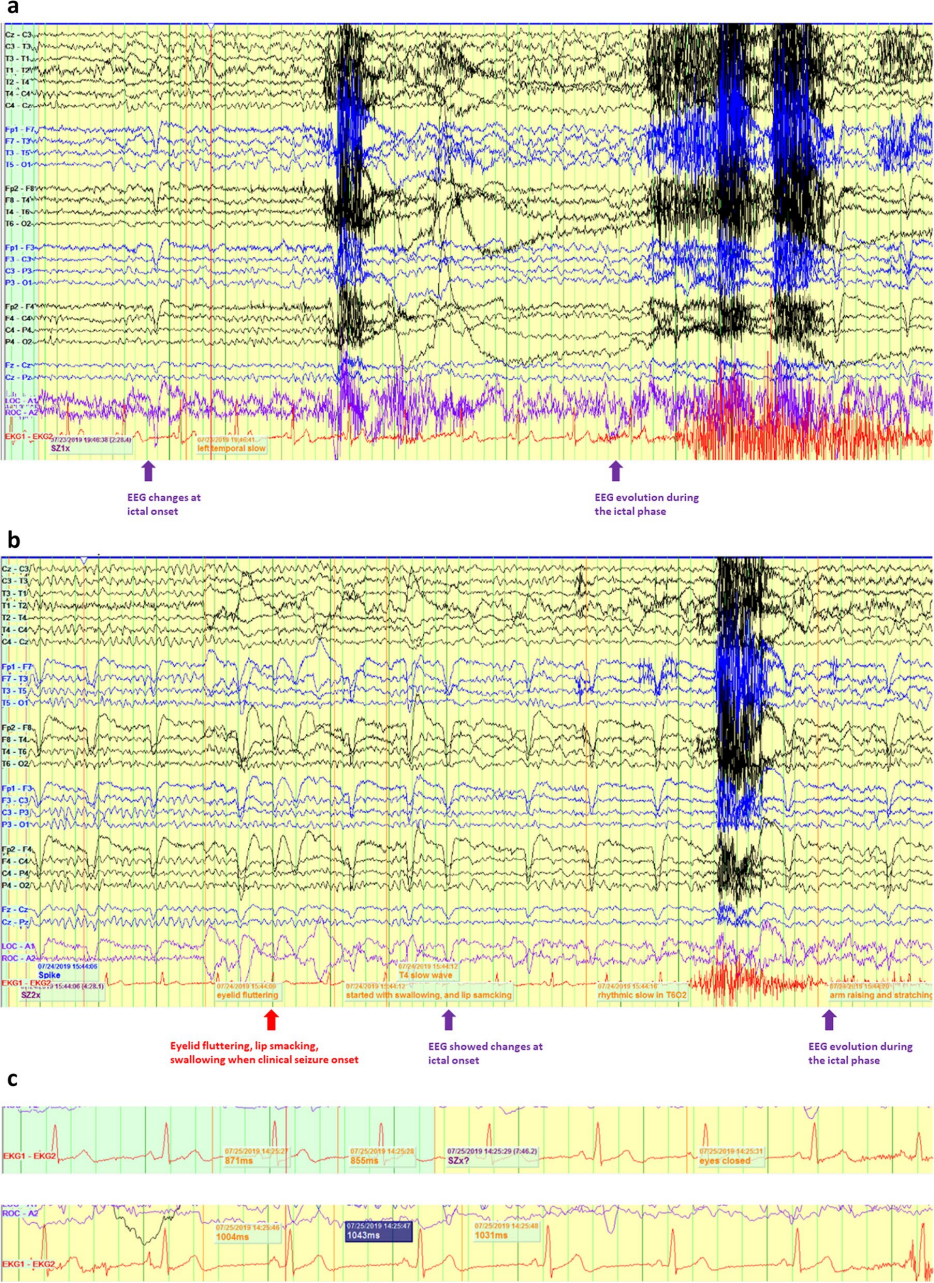
**a** A focal-onset seizure arising from the left temporal region. Purple arrow shows EEG focal slowing in the left temporal region at ictal onset and when ictal phase evolution. **b** A focal-onset seizure arising from the right temporal region. Purple arrow shows EEG focal slowing in the right temporal region at ictal onset and when ictal phase evolution, and red arrow indicates onset of clinical symptoms. **c** Ictal prolongation of RR interval during the focal-onset seizures, as recorded by the concurrent ECG monitoring. Blue arrow indicates the cardiac pacemaker pacing artifacts

Repeated vEEG two months after the pacemaker implantation showed interictal sharp waves in the bilateral temporal regions independently. Temporal intermittent rhythmic delta activity (1–2 Hz) was seen in the right temporal regions. During re-monitoring, seizures of bilaterally independent temporal onset were recorded (Fig. 2). Clinically, while being awake, the patient demonstrated staring or glassy eyes, with subsequent repetitive swallowing with or without lip smacking, and ictal coughing. During the events, the patient did not lose consciousness, but he was unable to maintain conversation and his wife reported that the patient did not make meaningful conversation; such observed semiology was similar to his habitual seizures before developing tonic and atonic seizures. The patient himself was amnestic to the events. At the onset of the seizures, EEG showed focal (irregular or semi-rhythmic) delta slowing in both temporal regions (Fig. 2 a, b). Subsequently, the ictal pattern evolved into diffuse semi-rhythmic delta-theta slowing. The ECG RR interval was analyzed at a baseline with heart rate of 62–63 bpm. During his seizure events, the RR interval increased by 100–200 ms, which triggered the pacemaker (Fig. 2c). The prolongation of RR interval often occurred 10–20 s after the onset of clinical seizures. This observation demonstrated that cardiac pacing prevented the development of ictal bradycardia/asystole during the patient’s focal seizures.

**Fig. 3:**
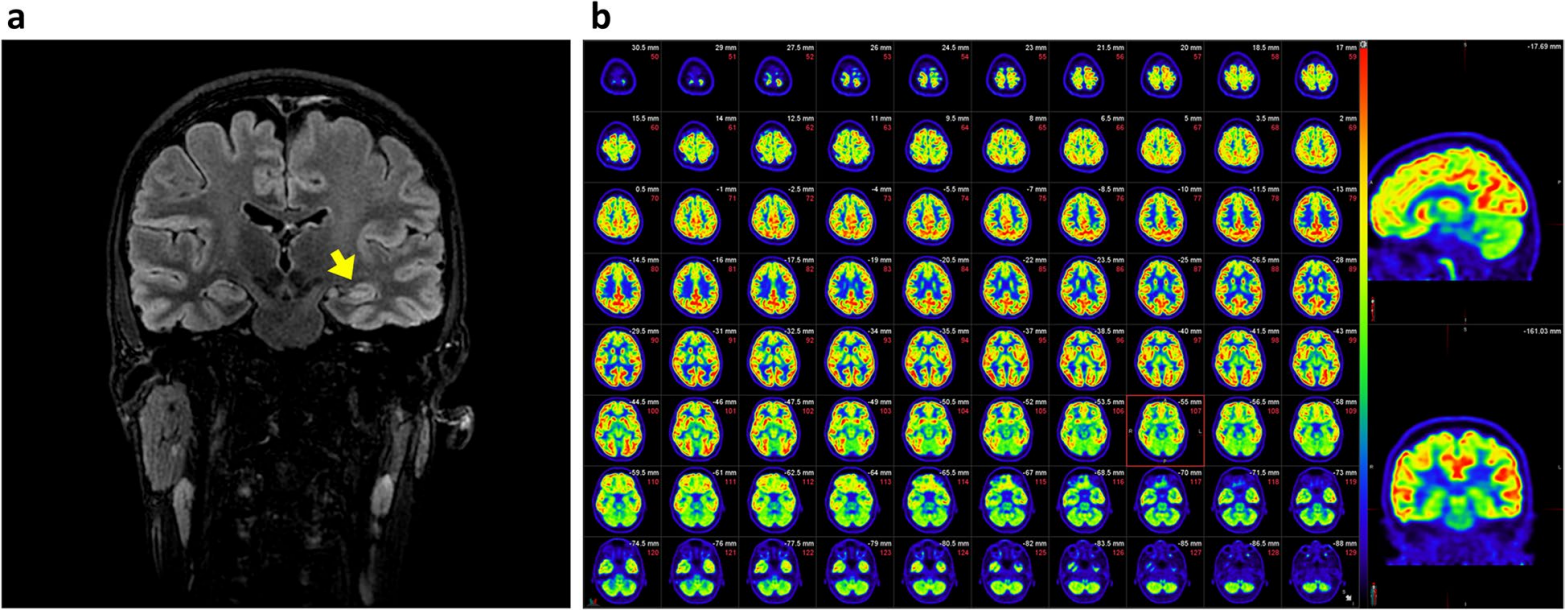
**a** Brain MRI showed left temporal and hippocampal sclerosis in the last follow up. Yellow arrow shows the increased T2/FLAIR signal and volume loss in the left hippocampus. **b** Brain PET showed no lateralized hypometabolism

Follow-up 3 T MRI showed left mesial temporal sclerosis with hippocampal volume loss and mild increase of FLAIR signal (Fig. 3a). Brain PET scan did not show lateralized hypometabolism (Fig. 3b).

The patient was followed up for three years, and no recurrent tonic or atonic seizures were reported. He continued with rather sporadic dyscognitive seizures while taking both Levetiracetam and Lamotrigine, Lacosamide was discontinued because no obvious effects were noted.

### Genetic testing and variant analysis

**Fig. 4:**
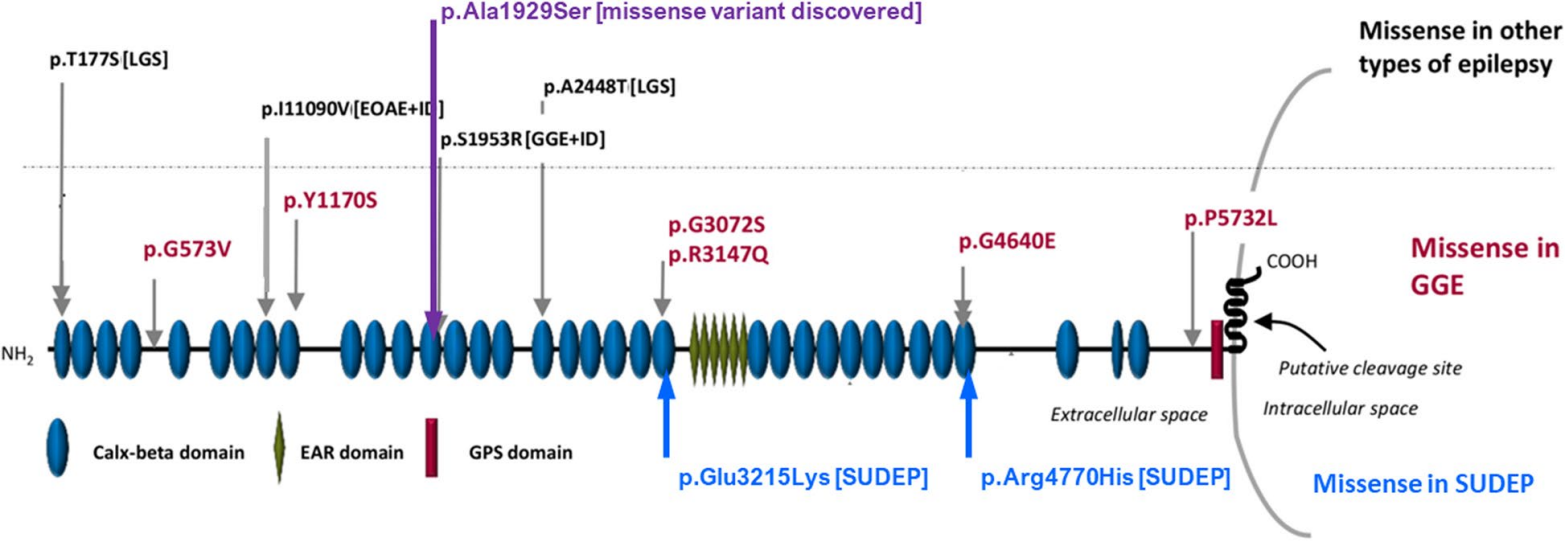
*ADGRV1* variants associated with epilepsy and SUDEP. Red,gene variants associated with genetic generalized epilepsy (GGE); black, variants associated with other type of epilepsy; blue, reported variants associated with SUDEP; purple, the de novo variant reported in our case. LGS: Lennox-Gastaut Syndrome; EOAE: early-onset absence epilepsy; ID: intellectual disability; EAR: epilepsy-associated repeat; GPS: G-protein-coupled receptors (GPCR) proteolytic site

Genetic analysis was conducted by using the next-generation sequencing for DNA sequence variants and copy number variants. Variants in dihydropyrimidine dehydrogenase (*DPYD*, c1905 + 1C > A), adhesion G protein-coupled receptor V1 (*ADGRV1*, c.5785 G > T) and two other variants were discovered (Table 1). These variants were not revealed in his parents. We performed in silico analysis with polyphen-2, SIFT, Grantham score, and Mutation Taster softwares to estimate if the variants were pathogenic or had a damaging functional effect (Table 1). We also applied Alphafold, an artificial intelligence system, to investigate whether the discovered variants would affect the protein structures. Results showed that the *ADGRV1* variant at the 5q14.3 chromosomal locus, was likely to have an impact on the protein structure. The discovered de novo variant in ADGRV1 was localized in the calx-beta domain within the very close vicinity to reported genetic loci associated with generalized epilepsy (Fig. 4).

**Table 1:** Results of genetic variant analysis

Gene name	Base change	Codon change	Variant type	Inheritance	Polyphen-2	SIFT	Mutation Taster Prediction	Grantham	Protein domain	Clinical relevance	Reported clinical symptoms associated with previously reported gene variants
*DPYD*	c.1905 + 1G > A	n/a	Heterozygous splice site	Autosomal recessive	n/a	n/a	Disease-causing	n/a	n/a	Pathogenic	Seizures, microcephaly, muscular hypotonia, developmental delay, and sensitivity to 5-FU toxicity
*ADGRV1*	c.5785G > T	p.Ala1929Ser	Heterozygous missense	Autosomal	Damaging	Tolerated	Disease-causing	99	Calx:beta 13	Likely pathogenic vs VUS	Febrile and afebrile seizures, focal epilepsy and SUDEP, Lennox-Gastaut syndrome, myoclonic epilepsy, Usher syndrome
*DYNC1H1*	c.11894C > T	p.Ser3965Phe	Heterozygous missense	Autosomal recessive	Benign	Not Tolerated	Disease-causing	155	n/a	Variant of unknown significance	Intellectual disability, malformations in cortical development, West syndrome, epileptic encephalopathy with continuous spikes and waves during slow sleep, spinal muscular atrophy, and Charcot-Marie-Tooth syndrome
*ASPM*	c.6711C > A	p.Asn2237Lys	Heterozygous missense	Autosomal dominant	Benign	Tolerated	Polymorphism	94	IQ repeat region	Likely benign	Intellectual disability, primary microcephaly

*Abbreviations*: *polyphen-2* Polymorphism Phenotyping V2, *Grantham* Grantham scores for conservative, *SIFT* Sorting Intolerant From Tolerant, *DPYD* dihydropyridine dehydrogenase, *ADGRV1* adhesion G protein-coupled receptor V1, *DYNC1H1* dynein cytoplasmic 1 heavy chain 1, *ASPM* assembly factor for spindle microtubules, *VUS* Variant of unknown significance

## Discussion

The case presented in this report reflects the complicated nature of ictal asystole in the context of evolving seizure semiology. Before the extensive epilepsy examinations, the patient did not show predisposing factors, and had no family history of cardiac arrhythmia or sudden unexpected death. Our report highlights the importance of vEEG-ECG monitoring in confirming the diagnosis of ictal bradycardia/asystole [8]. Brain MRI indicated left mesial temporal sclerosis in this patient. EEG re-monitoring after the cardiac pacemaker implantation also showed focal seizures likely arising from the left or the right temporal region. These findings are consistent with literature that reported ictal asystole occurring in temporal lobe or insular epilepsy, especially in patients with DRE [9]. However, there are no obvious associations between lateralized epileptogenesis and autonomic nervous system changes manifested as ictal bradycardia/asystole. The underlying mechanisms of the correlation between temporal lobe seizures and the ictal asystole may be related to the stimulation of the insula, the cingulate cortex, the amygdala, and the hypothalamus during such seizures, which may provoke asystole via autonomic control [9]. Being secondary to the epileptic seizure, the ictal bradycardia/asystole results from the disruption of normal cardiac rhythm caused by heart rate changes. Clinically, the affected patients likely manifest unexpected collapse or fall in later phases of seizures, as presented in our case. The changes of seizure semiology with tonic, atonic, or nocturnal brief myoclonic features displayed by the patient may be clinical manifestations of cerebral hypoperfusion due to ictal bradycardia/asystole.

Patients with intractable drug-resistant focal epilepsy are at a higher risk of ictal asystole, which may lead to SUDEP. Besides GTCS, ictal asystole associated with DRE has also been considered as a potential mechanism for SUDEP and has received increasing attention [10]. Although ictal asystole is rare, its associated red flag symptoms such as atonia and unexplained falls often lead to detrimental, life-threatening complications as seen in our case, resulting in intracranial hemorrhage and cardiac arrest. Thus, cardiac pacemaker implantation is advised for patients with documented ictal asystole and would often be a life-saving practice [10].

Understanding the precise etiology of epilepsy is the basis for precision medicine and tailored treatment for patients with epilepsy. Further exploring the genetic basis of epilepsy may lead to an improved understanding of the epileptogenesis, personalized medical management, and ultimately improved seizure control/prevention outcomes and quality of life [11]. In our case, genetic analysis revealed a pathogenic variant in *DPYD*, a likely pathogenic variant in *ADGRV1*, and a variant of uncertain significance in *DYNC1H1*. Identifying genetic markers associated with phenotypes has remained challenging due to the complexity of the human genome, the extreme polygenicity, and lack of reports for rare gene variants [12]. In our case, the patient did not present with phenotypes that have been reported in literature, such as severe epilepsy syndrome, microcephaly, intellectual disability, cortical malformation, or neuromuscular symptoms (Table 1). Instead, he had unique presentations of ictal asystole and temporal lobe seizure, and promising treatment outcomes, which prompted us to further investigate the underlying pathophysiology. With careful inspection of the genotype–phenotype association and comprehensive prediction of functional impact of variants, we found that the de novo variant in *ADGRV1* is very likely to be the cause of the patient’s ictal asystole. ADGRV1 is a large calcium-binding protein widely expressed in the central nervous system. The *ADGRV1* gene is an epilepsy-associated gene (Table 1) located at the 5q14.3 chromosomal locus, a site that has been previously reported to be associated with myoclonic epilepsy due to haploinsufficiency. *ADGRV1* is recently discovered to be strongly associated with SUDEP [13]. We hypothesized that the discovered missense variant might lead to dysfunction of ADGRV1 protein, contributing to the unique phenotypes, temporal lobe epilepsy and ictal asystole observed in our patient. Recent animal studies showed that ADGRV1 is required for the development of γ-aminobutyric acid (GABA)ergic interneurons. It is possible that the disruption of ADGRV1 function may result in dysfunction of cortical GABAergic neurons, which serves as a potential epileptogenic mechanism in humans [14]. Intriguingly, the discovered pathogenic variant in *DPYD* in our patient did not appear to be associated with the severe clinical phenotypes reported in literature. DPYD plays an important role in the metabolism of the antineoplastic agent 5-fluorouracil (5-FU) and patients with DPYD deficiencies can have severe cardiac toxicity [15]. A previous paper reported that 5-FU administration in a patient with DPYD mutation led to Takotsubo cardiomyopathy due to autonomic imbalance. This raises the possibility that DPYD variants may be a predisposing factor for autonomic dysfunction [15].

Although the precise causes for the variants remain to be further investigated, our report calls for increased recognition of and access to genetic testing for adult epilepsy patients. Phenotypic and clinical information are often critical to interpreting the significance of the discovered genetic variants, which improves the quality and accuracy in reclassifying the VUS [16].

## Conclusions

The diagnosis of ictal asystole requires long-term vEEG-ECG monitoring. Treatment after the confirmed diagnosis should be aimed at preventing recurrent seizures. The implantation of a pacemaker is recommended when the risk of SUDEP is deemed to be high based on observed or potential cardiac rhythm disturbances. Continuing pharmacological treatment is important, and further monitoring after pacemaker implantation can provide critical information such as the lateralization of the seizure onset in our case.

Ictal asystole and SUDEP are rare, and the underlying pathophysiological mechanisms remain elusive. The genetic basis of epilepsy is increasingly explored, which will encourage the possibility of precision treatments for specific genetic etiologies [11]. Our genetic testing and analysis suggested that the *ADGRV1* gene is a contributor to SUDEP. Such findings not only draw attention to the mutation spectrum of the *ADGRV1* gene, but also inspire genetic testing and early identification of patients with epilepsy who are at a high risk of SUDEP.

## Data Availability

The datasets analyzed during the current study are available from the corresponding author on reasonable request.

## Abbreviations

ADGRV1: Adhesion G protein-coupled receptor V1
DPYD: Dihydropyridine dehydrogenase
DRE: Drug-resistant epilepsy
EEG: Electroencephalogram
GTCS: Generalized tonic–clonic seizure
SUDEP: Sudden unexpected death in epilepsy patients
